# Rotational ‘cooling’ and ‘heating’ of OH^+^(^3^Σ^−^) by collisions with He: quantum dynamics revealing propensity rules under ion trap conditions^†^

**DOI:** 10.1080/00268976.2018.1442597

**Published:** 2018-03-06

**Authors:** L. González-Sánchez, R. Wester, F.A. Gianturco

**Affiliations:** a Departamento de Química Física, University of Salamanca, Salamanca, Spain; b Institut für Ionenphysik und Angewandte Physik, Universitaet Innsbruck, Innsbruck, Austria

**Keywords:** Rotationally inelastic dynamics, cooling rates in traps, quantum scattering calculations

## Abstract

Multichannel scattering calculations are presented for the low-energy collisions of the OH^+^ cation and He atoms, using an *ab initio* evaluation of the interaction potential, which had been obtained in earlier work, and a time-independent, multichannel treatment of the quantum dynamics carried out in this study using our in-house scattering code ASPIN. Given the presence of spin-rotation coupling effects, within an essentially electrostatic formulation of the interaction forces with He atoms in the trap, the ensuing propensity rules which control the relative size of the state-changing cross sections and of the corresponding inelastic rates, also computed at the most likely temperatures in an ion trap, are presented and analysed in detail.

## GRAPHICAL ABSTRACT

**Figure:**
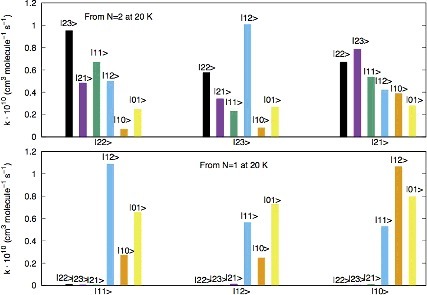

## 1. Introduction

The last 20 years or so have witnessed an outstanding progress in the experimental preparation of atoms and molecules, neutral and ionised, into confined environments with temperatures often down to the nanokelvin regimes where the manipulation of the target species into selected internal states has followed their storage at very low translational temperatures [1,2]. The range and breadth of the possible directions of enquiry onto the properties and behaviour of such prepared species has really been surprising since it has provided a very versatile tool for studying novel quantum systems [3], new quantum logic applications [4], ultra precise atomic clocks [5] and the possibility of controlling at low temperatures the behaviour of chemical reactions [6]. Hence, the maturing of these new techniques has rapidly triggered many applications to increasingly newer atomic and molecular systems for which one can investigate the properties at the molecular level.

One of the most successfully employed techniques has involved the process of translational and internal cooling of molecular ions by buffer-gas methods [7,8], whereby cold gas atoms are uploaded into the trap that contains the confined molecular partners in order to dissipate their translational and rotational energy down to the low temperature of the buffer gas selected (usually cold He atoms).

Due to the wide applicability of this technique, one can reasonably expect the buffer-gas cooling approach to successfully manage to thermalise the molecular rotor's states down to the low temperature experimentally reached by the buffer gas into the ion trap [9].

In a recent study carried out in our laboratory, for instance, the buffer-cooling technique has been applied to produce cold molecular ions of the OH^−^(^1^Σ) down to about a few K of rotational temperature (e.g. around 10–15 K) by using He as the uploaded gas [10]. The prepared target anions in a specific rotational state were further interrogated by a laser to photodetach the excess electron and selectively produce different rates of formation of neutral final products, depending on the initial rotational state population of the anion. Thus, the selective preparation of the anionic molecules in the trap could be successfully monitored via the photodetachment process, the latter being further modelled by collisional quantum dynamics calculations carried out at the experimental conditions [10].

Similar *ab initio* quantum studies, which are linked with ongoing photodetachment experiments in our laboratory, have been recently completed for the polyatomic anion of NH^−^_2_(^1^ *A*_1_), also interacting with He as a buffer gas [11]. They have verified by calculations the possibility that to selectively photodetach the excess electron from an internally prepared molecular anion in a cold trap can lead to detectable relative differences in the corresponding rates at low temperatures.

Another ionic system of current interest is the corresponding cation of the hydroxyl molecule: the OH^+^(^3^Σ^−^) confined in a cold ion trap and made to interact with He as the uploaded buffer gas. We have studied earlier on this system in order to obtain the *ab initio* potential energy surface (PES) for its interaction with the He atom [12] and then use it to further obtain the dynamical behaviour down to the nanokelvin regime [13]. In a recent analysis of the same system [14], we have studied its collisional features in ion traps by using a more recent version of its PES and by carrying out the inelastic cross section calculations within the recoupling approximation of its spin-rotation structure already applied earlier by us to another cationic molecule [15].

In the present investigation, we wish to extend the study by fully including the additional effects on the dynamics from the spin-rotation angular momentum coupling and extract from quantum calculations the observability of specific propensity rules governing the relative sizes of the state-changing rotationally inelastic rate constants at the expected temperatures of typical experiments in cold ion traps (e.g. from 10 to about 50 K).

Such a molecule is also of great interest for its role on the chemistry of light hydrides in the Interstellar Medium (ISM), where the rotational line emission of OH^+^ has been detected, among other places, in planetary nebulae hosting hot central stars [16] and toward the Supernova associated with the Crab Nebula [17].

In what follows, we shall concentrate on its collisional behaviour for rotationally inelastic processes induced by the uploaded He gas in the cold ion trap and we shall describe in some detail the interplay of the various dynamical angular momentum couplings in generating excitation and relaxation rates at the low temperatures of interest here.

Section 2 briefly describes the main features of the employed PES and the structure of the energy levels involving the (^3^Σ) open-shell target. Section 3 will outline the calculations and the results obtained for the angular momentum coupling cross sections and will also present and discuss the corresponding inelastic rate constants obtained up to about 50 K, to provide a simple modelling of the collision-driven population kinetics in the trap. Our present conclusions will be summarised in Section 4.

## 2. Interaction forces and rotor's structure

The spatial anisotropy of the electronic forces acting between the polar, ionic target molecule and the He(^1^ *S*) atoms uploaded in the trap is a crucial piece of information for assessing the efficiency of the state-changing of that target's rotational levels by collision with neutral atoms. Specifically, the angular dependence of these forces in the [*R*, θ] Jacobi space is a direct indicator of that efficiency, since the torque applied to the molecular rotor during collisions strongly depends on that anisotropy. Years ago [12,13] we had calculated the V(R,θ) interaction at the post-Hartree–Fock level by using the MP4 formalism with a basis set expansion of the aug-cc-pVQZ quality and a detailed inclusion of the basis-set-superposition-error correction via the counterpoise method [18]. The overall range of values covered 320 radial points over 12 values of the θ angle between 0° and 180°. The fitting procedure followed by us to generate an analytic form of the V(R,θ) potential was also described earlier [12,13] and will not be repeated here.

A later study [19] considered the larger Jacobi space of V(R,r,θ) which also included dependence on the vibrational coordinate of the cation. The employed basis set expansion was also an aug-cc-avQZ and used the CCSD(T) expansion method as described in [20] and implemented by the MOLPRO suite of codes [21].

A comparison between the behaviour of the above two PESs in terms of their multipolar coefficients for an expansion taken at the molecular equilibrium geometry, r_eq_=1.0279 Å,

(1) $$\begin{array}{c} V\left(R,\theta |r_{\text{eq}}\right)=\sum_{\lambda =0}^{\lambda_{\text{max}}}V_{\lambda }\left(R|r_{\text{eq}}\right)P_{\lambda }\left(\cos\theta \right) \end{array}$$

was recently carried out by us [14] and found that only minor differences existed between the two sets of coefficients. For the sake of consistency, we have now employed the more recent PES results from [19] to carry out the present calculations.

**Figure 1.**
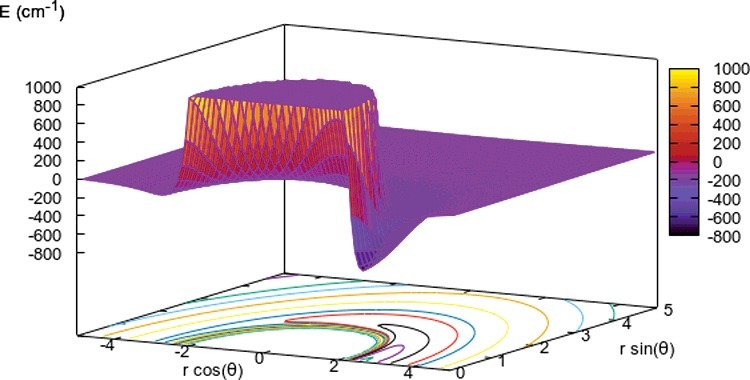
3D view of the computed V(R,θ) PES for the r_eq_ molecular bond length. The on-plane projection of the isoenergy curves is shown at the bottom of the figure. The OH^+^ molecule is along the rcos (θ) axis, with the origin of that axis at the center-of-mass (com) of the molecule and the H atom along the positive branch of the axis.

A pictorial view of the orientational anisotropy of the employed PES is presented in the data of Figure 1.

One clearly sees, qualitatively speaking, the marked attractive well on the H side of the molecule and the overall repulsive region around the space of the molecular bond. We discussed before the relative strength of the multipolar coefficients in Equation (1) [14], indicating there the dominant effect from the λ = 1 coefficient over a large region of the radial interaction. Such feature will be clearly revealed by the present calculations and was already discussed in ref. [14]. The long-range (LR) shape of the PES was also reported there to be dominated by the He atom dipole polarisability and the permanent dipole moment of the OH^+^ partner:

(2) $$\begin{array}{c} V_{\text{LR}}\left(R,\theta \right)∼\frac{\alpha_{0}}{2R^{4}}+\frac{2\alpha_{0}\mu }{R^{5}}\cos\theta +\cdots \end{array}$$

### 2.1. Rotational structure of OH^+^(^3^Σ)

The open-shell target rotor was treated as a pure Hund's case (b), so that the total dynamic's angular momentum is **J** = **N** + **S** with **N** being its total rotational angular momentum and **S** the total electronic spin angular momentum. Furthermore, since the hydrogen atom possesses a non-zero nuclear spin (**I** = 1/2), an additional coupling occurs and another quantised angular momentum **F** has to be introduced, the latter resulting from the coupling of **J** with **I**: **F** = **J** + **I**.

On the other hand, the above hyperfine splitting of the rotational levels induced by the nuclear spin effects is much smaller than the splitting induced by the spin-rotation couplings from the electronic structure. It will therefore be treated as degenerate in the present study, where the (**N**,**S**) couplings will be however explicitly considered:

(3) $$\begin{array}{ccc} |F_{1}\;J\;M\rangle & = & |N=J-1,S\;J\;M\rangle \\ |F_{2}\;J\;M\rangle & = & |N=J,S\;J\;M\rangle \\ |F_{3}\;J\;M\rangle & = & |N=J+1,S\;J\;M\rangle \end{array}$$

where *M* is the projection of **J** along the space-fixed (SF) axis and the F_*i*_s are the splitting-induced components of the total angular momentum **N**.

**Figure 2.**
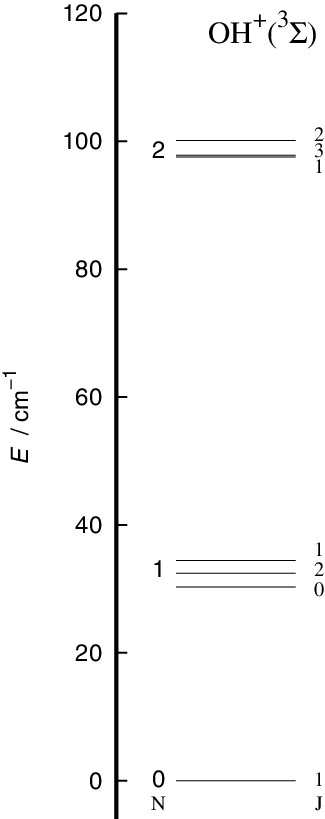
Computed energy level splittings for the lowest three levels of the OH^+^(^3^Σ) rotor considered in the present study. The (**N**,**S**) coupling effects are explicitly shown, while the hyperfine splittings from (**J**,**I**) coupling have been omitted because of their smallness.

The spacing in energy between the lower rotational levels of the open-shell rotor are reported by Figure 2, where the effect of the nuclear-spin splitting, already shown to be negligible, has been omitted. We have used the experimental spectroscopic constant from [22] on the energy level spacing as defined earlier in [13].

## 3. Coupled-channel quantum dynamics

If we disregard from the moment the further hyperfine labelling due to the couplings from the **F** angular momentum, we will then be including the fine coupling effects between **I** and **J** mentioned earlier. Hence, we simply consider the level splitting due to the spin-rotation couplings from the pure Hund's case (b) and therefore describe the collision of the cationic molecule in a particular |*F*_*i*_ *JM* > rotational state from Equation (4) with the structure-less He partner by expanding the total wavefunction in terms of the eigenfunctions of the total angular momentum J, with SF projection onto the *z*-axis given by M [13,23,24]:

(4) $$\begin{array}{c} |F_{i}JLJM\rangle =\sum_{MM_{L}}\langle JMLM_{L}|JM\rangle |F_{i}JM\rangle \end{array}$$

where <....|.. > is a Clebsch–Gordan coefficient [13], *L* is the relative motion's orbital momentum quantum number and |*LM*_*L*_ > the wavefunction for the rotational motion of the He atom. The label *F*_*i*_ refers to the spin-rotation splitting given by Equation (4). This can also be written as

(5) $$\begin{array}{c} |F_{i}JM\rangle =\sum_{N=J-1}^{N=J+1}c_{NF_{i}}^{J}|NSJM\rangle \end{array}$$

By solving the coupled-channel (CC) equations [23], we can obtain from their asymptotic behaviour the matrix elements of the scattering Matrix **S** labelled by J plus the indices of the initial and final rotational states of the molecular cation [24]. By using the general expansion 5 one can obtain the transition amplitudes involving the rotational wavefunctions of the pure Hund's case (b) given in 4. The corresponding transition matrix **T** is given as: **T** = **1** – **S**.

Independently of the choice of the quantisation axis, one can further define the degeneracy averaged, inelastic state-to-state integral cross section as [24]

(6) $$\begin{array}{c} \sigma_{\left|NJ>\to \right|N^{'}J^{'}>}=\frac{\pi }{\left(2J+1\right)k_{NJ}^{2}}\sum_{K}P_{NJ\to N^{'}J^{'}}^{K} \end{array}$$

where $P_{NJ\to N^{'}J^{'}}^{K}$ is the tensor opacity given by

(7) $$\begin{array}{c} P_{NJ\to N^{'}J^{'}}^{K}=\frac{1}{2K+1}\sum_{LL^{'}}{\left|\langle N^{'}J^{'}L^{'}|T_{K}|NJL\rangle \right|}^{2} \end{array}$$

where *N* is defined in Equation (3) and each of its values corresponds to a different *F*_*i*_ value. Here *K* varies between (*J* + *J*′) and |*J* – *J*’| and *L* is the orbital angular momentum quantum number, with the sum being over the matrix elements of the **T** matrix operator.

It has been shown earlier [24,25] that the opacity tensor can also be rewritten as

(8) $$\begin{array}{c} \sum_{K}P_{NJ\to N^{'}J^{'}}^{K}=\sum_{K}\left\{\begin{array}{ccc} N & N^{'} & K\\ J & J^{'} & S \end{array}\right\}P_{N\to N^{'}}^{K} \end{array}$$

where $\left\{\begin{array}{ccc} \cdot & \cdot & \cdot \\ \cdot & \cdot & \cdot \end{array}\right\}$ is a 6-j symbol and the transitions between the |*N*> levels of Figure 2 is expressed in terms of angular momentum recoupling coefficients involving the physical rotational states of the target and its electronic spin angular momentum values. It will help us in the following discussion to point out the physical origins of the propensity rules which control the relative sizes of the inelastic cross sections obtained for the present system.

The scattering calculations were carried out using our own code ASPIN [23] and in those calculations we coupled all channels from *N* = 1 to *N* = 5. Several closed channels were included, depending on the collision energy, to ensure numerical convergence of the final inelastic cross sections. The range of integration was extended at the lowest energies out to 300 Å and at least 10 closed channels were included in the expansions. The expected numerical convergence of the cross sections is within 0.1% of their values.

### 3.1. The inelastic cross sections

One should be aware of the fact that only electrostatic forces are involved in the interaction potential described in Section 2. They are acting directly, through their anisotropic features, on the dynamical torques which drive the rotational state-changing collisions. On the other hand, the spin–flip processes which involve changes of the orientation of the spin orbital angular momentum **S** are only caused indirectly via the reorientation of **N** after **J** has changed. Thus, no direct physical interactions affect spin changes during the collisions. This feature of the dynamics will therefore guide the propensity rules which act on controlling the relative size of the inelastic cross sections that we shall be discussing in the following.

**Figure 3.**
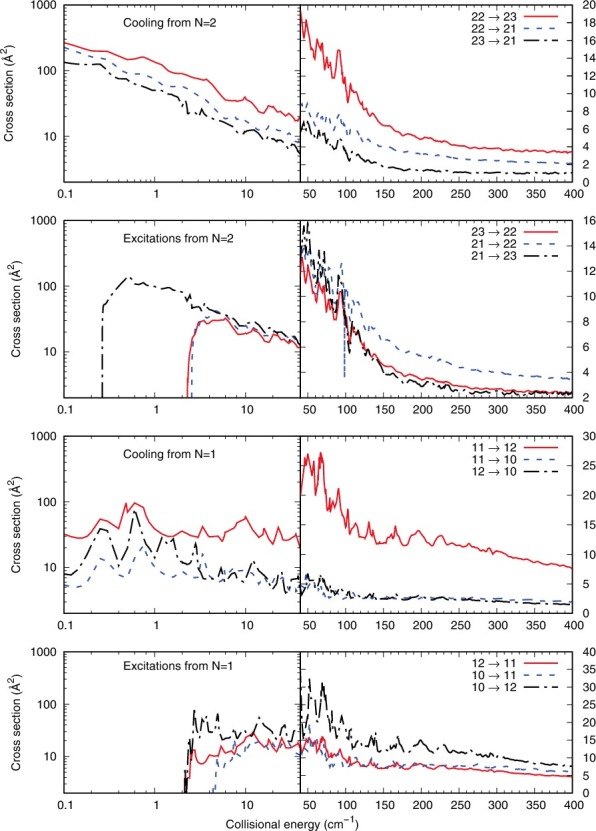
Computed inelastic cross sections for the Δ*N* = 0 processes, whereby only the |*J*> and |*S*> quantum numbers are changed. See main text for further details.

The panels of Figure 3 present the cooling and excitation cross sections from the lowest two |*N*> excited levels which we have considered and that would be the more likely to be involved in trap experiments. All the cases presented in that figure treat transitions from which Δ*N* = 0, i.e. we observe inelastic cross sections between different |*J*> levels, where the spin angular momentum is therefore also realigned to follow the Δ*N* = 0 constraint.

In the lower range of collision energies, we see that all cross sections correspond to nearly elastic processes, with very small energy transfer values and only with changes of the |*J*> and |*S*> quantum numbers according to Equation (5). Hence, all cross sections are very large and rapidly decrease with increasing collision energy. We further see that the cross sections associated with positive values of the Δ*J* transitions, within each panel, are invariably yielding the larger cross sections. The 6-j symbol of Equation (8) provides for those processes the larger coefficients to the *P* ^*k*^ values.

**Figure 4.**
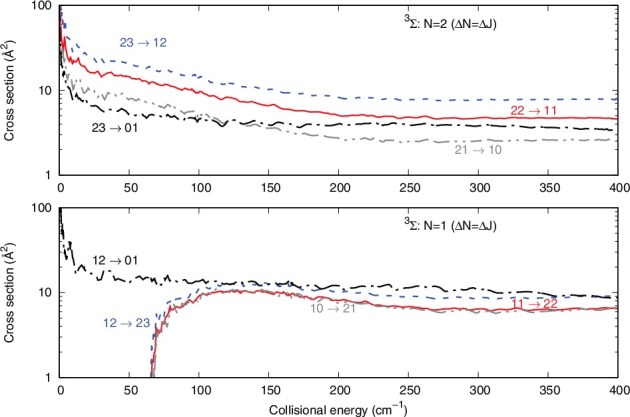
Computed inelastic cross sections for the Δ*N* = Δ*J* processes. See main text for further details.

The two panels of Figure 4 present a different set of situation, where Δ*N* is now different from zero and it is equal to Δ*J*. From Equation (8) we can see that such cross sections should in fact be the largest cross sections involving physical rotational transitions in the molecular ion.

The upper panel shows inelastic transitions form the |*N*> = 2 level, while the lower panel shows processes from the |*N*> = 1 level. In the former panel, the largest cross sections correspond to Δ*J* values which are associated with the largest |*J*> values between transitions: the role of the 6-j coefficients in Equation (8) indicates that more terms are present when |*J*> and |*J*′'> are the largest and *K* therefore contributes to more of them . The same type of effect is seen for the excitation process in the lower panel, where the (1,2)→(2,3) cross section in the largest. We further see in the upper panel that the increase of Δ*N* produces smaller cross sections compared with those where Δ*N* = Δ*J* and is equal to 1. This effect is obviously linked with the increasing of the energy gap, which is usually causing a decreasing of the cross section size.

**Figure 5.**
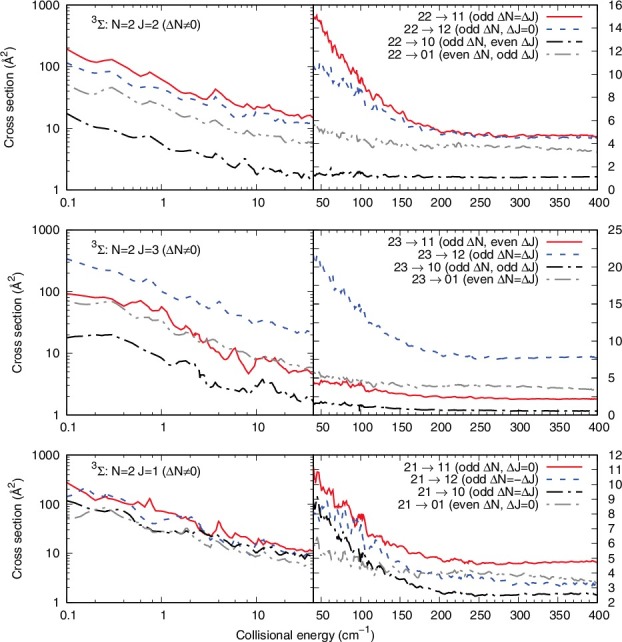
Computed inelastic cooling processes from the three different fine-structure levels of the |*N*> = 2 initial level of the ^3^Σ rotor. See main text for further details.

The effect of the propensity rules as depicted by Equation (8) could also be seen when comparing the results reported by the panels of Figure 5. What we report in that figure are the cooling transitions from the three fine-structure levels of the |*N*> = 2 initial state of the ^3^Σ rotor (see data in Figure 2). It is interesting to note that, in all three panels, the largest cross sections correspond to Δ*N* = Δ*J*, with the Δ*N* = 0 cross sections being also very close in size over a broad range of collision energies. Furthermore, situations where Δ*N* changes by 1 (odd Δ*N*) are also corresponding to larger cross sections than those obtained for the case of even Δ*N*, i.e. for Δ*N* = 2: it is again the increased energy gap which causes smaller inelastic cross sections to occur. Thus, both Δ*N* = Δ*J* and Δ*J* = 0 cross sections appear to be favoured transitions for the present system.

### 3.2. The computed state-changing rates

In order to model the kinetic behaviour in the trap, we need to further obtain the corresponding rates at the temperature of interest:

(9) $$\begin{array}{c} \;\;\;\;k_{NJ\to N^{'}J^{'}}\left(T\right)={\left(\frac{8}{\pi \mu k_{B}^{3}T^{3}}\right)}^{1/2}\int_{0}^{\infty }E\;\sigma_{\left(NJ\to NJ^{'}\right)}\left(E\right)e^{-\frac{E}{k_{B}T}}\;dE \end{array}$$

**Figure 6.**
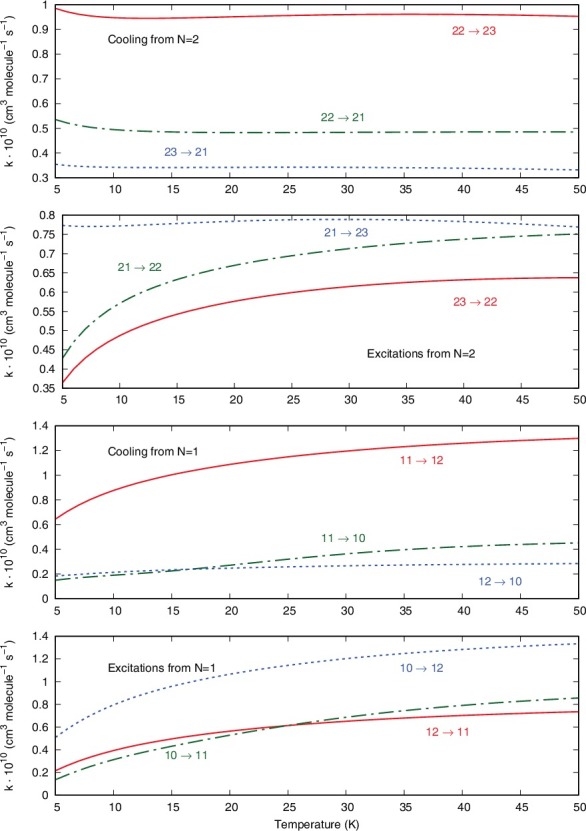
Computed inelastic rates for transitions involving only Δ*J* ≠ 0, with Δ*N* = 0 for all of them. See main text for further details.

The panels of Figure 6 report the cooling rates, over a range of temperatures from 5 to 50 K, from the |*N*> = 2 and |*N*> = 1 levels (top panel and second from bottom) together with the excitation rates from |*N*> = 1 (bottom panel) and |*N*> =2 (third panel from the bottom). All the processes considered conserve the |*N*> quantum number, thus indicating only Δ*N* = 0 inelastic rates.

One clearly sees that the dominant cooling processes are associated with Δ*J* = +1, both for the |*N*> = 2 and the |*N*> = 1 initial states. Furthermore, the excitation processes are also dominated by the Δ*J* positive values in all panels.

**Figure 7.**
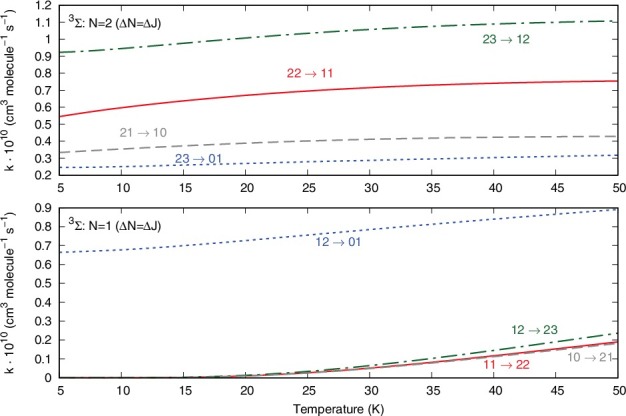
Computed inelastic rates from the |*N*> = 1 state (lower panel) and the |*N*> = 2 state (upper panel), showing only transitions with Δ*N* = Δ*J*.

The data of Figure 7 report now the excitation processes with Δ*N* = Δ*J*, starting from the |*N*> = 1 (lower panel) or the |*N*> = 2 (upper panel) states of the ^3^Σ rotor.

Since the corresponding cross sections were found to be the largest ones, we see here that the rates are also fairly large. Furthermore, the Δ*N* = Δ*J* = –2 transitions show smaller rates than those associated with Δ*N* = Δ*J* = –1.

**Figure 8.**
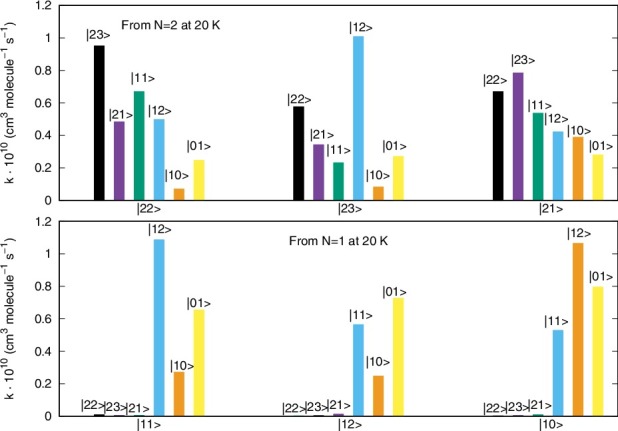
Computed relative values of different inelastic rates at a trap temperature of 20 K. See main text for further details.

A different pictorial way of showing propensity effects on the relative size of the state-changing rates is reported by the two panels of Figure 8.

The two panels of Figure 8 report the excitation/deexcitation processes occurring from the three sub-levels of |*N*> = 2 rotor state (upper panel) and from the sub-levels of |*N*> = 1 rotor state (lower panel). The following considerations could be made from a perusal of the results reported in that figure:

(1) the Δ*N* = 0 processes, physically involving a reorientation of the spin angular momentum without changes for the molecular rotational energies, seem to be the largest processes from the |*N*> level, associated with positive values of the Δ*J* changes;
(2) from the |*N*> = 2 level the Δ*N* = Δ*J* = –1 terms turn out to yield the largest rates at this temperature, followed by the Δ*N* = 0 processes. For the latter transitions, the positive Δ*J* changes cause larger rates than those with negative Δ*J* changes. Such rules are also linked to different values of the 6j-symbols appearing in Equation (8).

**Figure 9.**
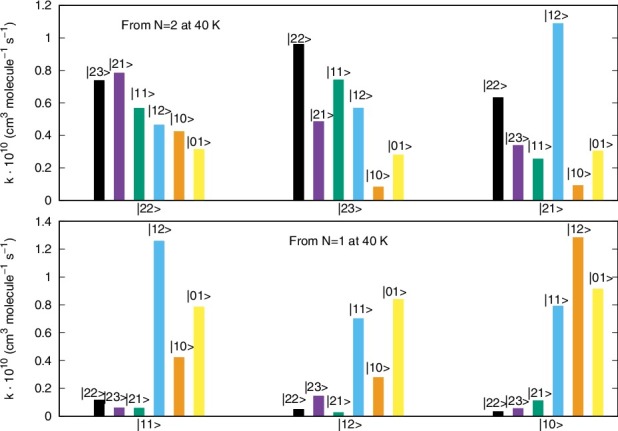
Same calculations as those of Figure 8 but referring here to a temperature of 40 K.

The data reported by Figure 9 examine the inelastic rates at the highest temperature which we have computed in the present study.

These reported cross sections do not show a marked dependence on temperature since the relative sizes of the rates reported in this figure are very similar to those computed at 20 K in Figure 8. Furthermore, we see in both panels the dominance in size of the Δ*N* = 0 processes, where little energy is being transferred and chiefly indirect spin realignment occurs during the transitions. Next to them, the Δ*N* = Δ*J* processes also dominate the rates, with a preference with situations where the Δ*J* value is a positive one. In case the experiments could separate the fine structure transitions for this system, then the present calculations suggest clear dominance of some specific transitions which should then become amenable to observation.

## 4. Present conclusions

In the present work, we have analysed in some detail the collisional state-changing processes for the open-shell rotor OH^+^(^3^Σ) at the low-temperature conditions of an ion trap and interacting with He(^1^S) as the buffer gas uploaded into the trap.

Using an accurate, *ab initio* calculation of the PES for the rigid-rotor target, we have carried out quantum, multichannel evaluations of a broad range of inelastic cross sections involving the |*N*> = 1, 2 states of the ^3^Σ rotor. Such processes are expected to be the relevant state-changing transitions for the experimental detection.

We also attempted to establish from the *ab initio* calculations the possible presence of propensity rules controlling the relative size of the inelastic cross sections and of the corresponding inelastic rates at temperatures from 5 to 50 K.

The following propensity rules could be extracted from the present quantum calculations:

(1) The Δ*N* = 0 transitions, chiefly involving spin reorientation processes, rather than physical rotational state changes, turn out to be among the largest cross sections and the largest rates at the considered temperatures;
(2) The Δ*N* = Δ*J* processes are also dominant among the transitions involving rotational excitation/deexcitation events. We also found, as was the case in previous work [19], that when Δ*N* increases the corresponding cross sections and rates become smaller;
(3) For transitions involving changes in the |*J*> angular momentum, we found that those involving positive values of Δ*J* are associated with larger transition probabilities.

The physical origin of the results we have found turns out to be similar to that analysed years ago [22,25] and to be linked to the properties of the corresponding 6j-coefficients appearing in Equation (8) and controlling the overall size of the opacity functions associated with them in generating the final cross sections.

Thus, to select specific transitions which can be detected in cold trap experiments [10] can be guided by the relative sizes of the different transitions that are suggested in the present study.
